# Plasma-Assisted Growth of Silicon Nanowires by Sn Catalyst: Step-by-Step Observation

**DOI:** 10.1186/s11671-016-1681-5

**Published:** 2016-10-12

**Authors:** Jian Tang, Jean-Luc Maurice, Wanghua Chen, Soumyadeep Misra, Martin Foldyna, Erik V. Johnson, Pere Roca i Cabarrocas

**Affiliations:** LPICM, CNRS, Ecole Polytechnique, Université Paris-Saclay, 91128 Palaiseau, France

**Keywords:** Silicon nanowires, Sn, PECVD, VLS, Growth process

## Abstract

A comprehensive study of the silicon nanowire growth process has been carried out. Silicon nanowires were grown by plasma-assisted-vapor-solid method using tin as a catalyst. We have focused on the evolution of the silicon nanowire density, morphology, and crystallinity. For the first time, the initial growth stage, which determines the nanowire (NW) density and growth direction, has been observed step by step. We provide direct evidence of the merging of Sn catalyst droplets and the formation of Si nanowires during the first 10 s of growth. We found that the density of Sn droplets decreases from ~9000 Sn droplets/μm^2^ to 2000 droplets/μm^2^ after just 10 s of growth. Moreover, the long and straight nanowire density decreases from 170/μm^2^ after 2 min of growth to less than 10/μm^2^ after 90 min. This strong reduction in nanowire density is accompanied by an evolution of their morphology from cylindrical to conical, then to bend conical, and finally, to a bend inverted conical shape. Moreover, the changes in the crystalline structure of nanowires are from (i) monocrystalline to (ii) monocrystalline core/defective crystalline shell and then to (iii) monocrystalline core/defective crystalline shell/amorphous shell. The evolutions of NW properties have been explained in detail.

## Background

Semiconductor nanowires (NWs) are highly desirable for new generations of electronic and photonic devices such as transistors [1], memory [2], biosensors [3], photodetectors [4], solar cells [5–8], and battery electrodes [9, 10]. Vapor-liquid-solid (VLS) is the most widely used method to synthesize NWs, as first demonstrated in the original work of Ellis and Wagner [11], and Au is the most widely studied metal catalyst for VLS growth [12–15]. However, Au has drawbacks when combined with the most popular semiconductor, silicon (Si), because Au atoms incorporated into Si induce deep level electrical defects [16, 17]. Moreover, it is eutectic with S, which determines the growth temperature of 363 °C, a temperature quite high for applications on flexible substrates. With the assistance of plasma, Sn can be used to synthesize less defective Si NWs at lower temperature, ~232 °C, by VLS. In addition, due to the low solubility of Si in Sn at the nanowire (NW) growth temperature, atomically sharp heterostructures can be achieved when using a Sn catalyst [18]. Obtaining a periodic array of ordered NWs should be possible if we were using nanopatterned catalysts on c-Si substrates [19]. Moreover, oriented growth with all the NWs growing perpendicular to the substrate has also been demonstrated for Si NW growth on <111> oriented c-Si wafers [20]. However, our main goal is to develop a low-cost process for Si NWs growth in view of their use as a support for radial junction solar cells [8, 21, 22]. In this approach, we use Al doped ZnO (ZnO:Al) deposited on glass as a standard substrate, which makes it difficult to obtain NWs with well-controlled growth direction, diameter, density, and crystalline quality. Thus, a better understanding of the growth process is essential. In the literature, only few papers dedicated to study the SiNW growth by plasma-assisted-vapor-solid method using tin as a catalyst can be found [23–29]. In these studies, different growth temperature [23], plasma power density [25], catalyst size [23, 28], and hydrogen dilution level [23, 24] have been used as variables to study the variations of the nanowire geometry and crystallinity. However, the growth process was not detailed in these studies. There are few papers which investigate the nanowire diameter and length evolution [24, 29] and discuss the growth process. However, the initial growth stage (when catalysts droplets form small NWs) was overlooked, despite the fact that this stage is the most important one for understanding the growth process. Indeed, the density and the orientation of the NWs as well as the diameter of the crystalline core are mainly determined within this stage. This growth stage is the focus of this work. In addition, we have expanded the duration of the NW growth time until the merging of NWs. Moreover, we also provide detailed statistical analysis and explanations on the evolution of NW density, morphology, and crystalline structure.

## Methods

In this work, the silicon NW growth experiment involves the following steps:

1. A ~1.4-μm thick ZnO:Al film was sputtered onto Corning glass. During the sputtering process, the substrate temperature was 200 °C [30].
2. A Sn layer with a nominal thickness of 1 nm was deposited on ZnO:Al by thermal evaporation. These droplets subsequently oxidize when exposed to atmosphere.
3. The substrate was loaded into a standard capacitively coupled plasma-enhanced chemical vapor deposition (PECVD) reactor and was heated up to 200 °C under vacuum.
4. A hydrogen plasma was applied to reduce the tin oxide and to form Sn droplets. The H_2_ flow rate, pressure, RF power density, interelectrode distance, and duration of the plasma were 100 sccm, 600 mTorr, 38 mW/cm^2^, 28 mm, and 2 min, respectively.
5. After the hydrogen plasma treatment, the temperature of the substrate holder was increased to 400 °C, and samples were annealed for 2 min under a H_2_ flow. The H_2_ flow rate and the pressure are same as in step 4. Then 11 sccm of silane was introduced. The total pressure for SiH_4_ and H_2_ gas was set to 1000 mTorr. RF power of 17 mW/cm^2^ was applied to trigger the silane plasma. The duration of NW growth was varied between 1 s and 8 h. After the growth, the samples were cooled down to room temperature under H_2_ flow.

The samples were systematically analyzed by scanning electron microscopy (SEM) and Raman spectroscopy. Transmission electron microscopy (TEM) was also carried out on selected samples. The SEM equipment was a Hitachi S-4800; the TEM instrument was a Jeol 2010F with a point-to-point resolution of 0.23 nm. The Raman spectrometer was an ARAMIS system from Jobin-Yvon with a lateral resolution around 1 μm and an excitation wavelength of 473 nm.

## Results and Discussion

### NW Growth Process

The NW growth process from catalyst formation to the end of growth (up to 8 h) has been systematically analyzed by SEM. In this paper, we roughly divide the whole growth process into four phases: (i) the evolution of catalyst droplets and initial formation of NWs, (ii) the formation of straight NWs, (iii) the NW growth, and (iv) the end of NW axial growth followed by a-Si:H coating.

#### Evolution of Catalyst Droplets and Initial Stages of NW Growth

**Fig. 1:**
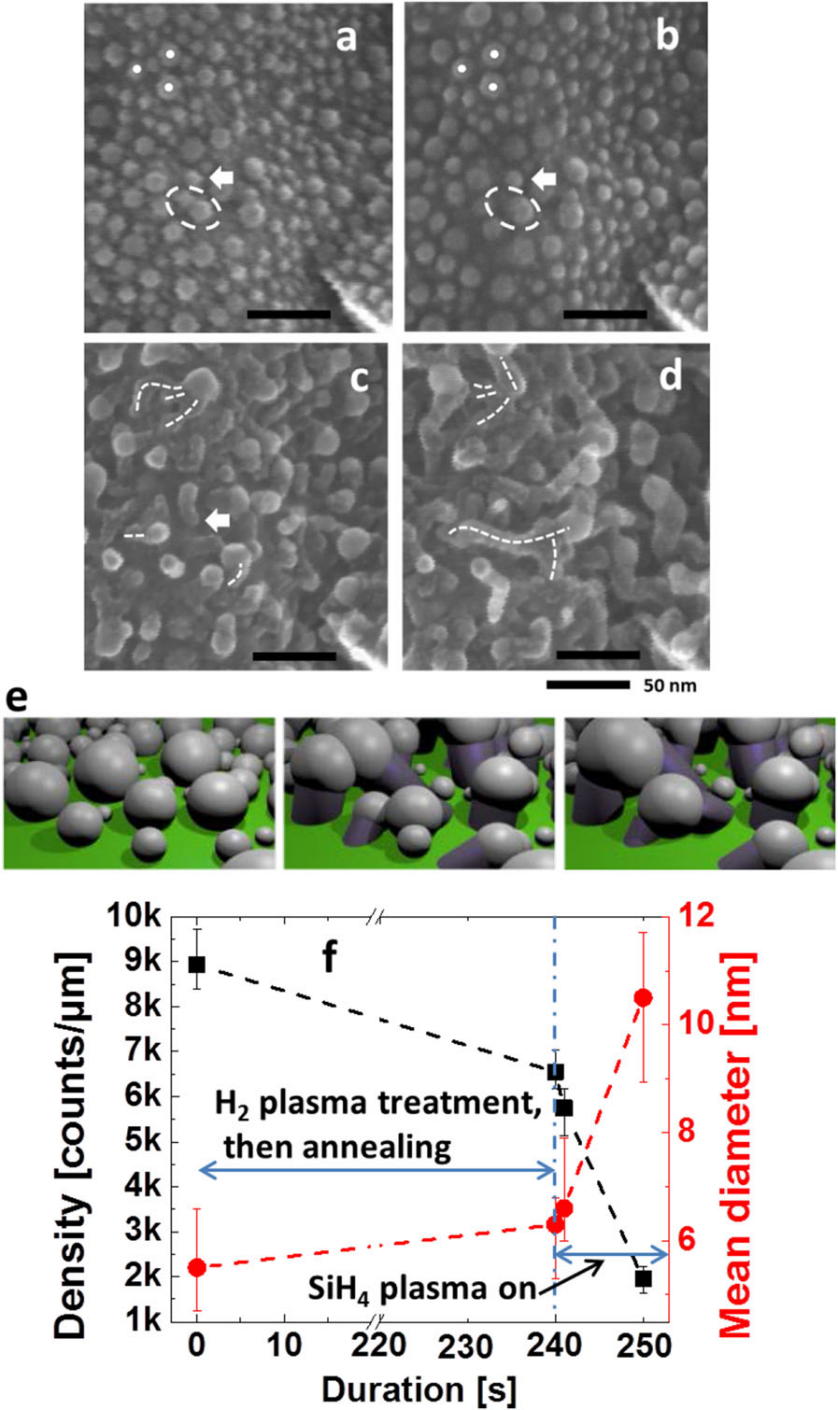
**a**–**d** SEM images acquired at the same spot on one sample at different growth times. **a** 1 nm of Sn deposited on ZnO:Al. **b** After 2 min of H_2_ plasma treatment at 200°C and 2 min of annealing at 400°C. **c** After 10 s of NW growth at 400°C. **d** 40 s growth. **e** 3D illustration of coalescence of droplets during the first 10 s NW growth. **f** Evolution of the droplets density and mean diameter as a function of process time

In order to precisely monitor the evolution of the catalyst droplets at the initial stages of SiNW formation, we have made a mark on the substrates (white stripe on the bottom left corner of the images) allowing us to return to the same location on the sample after each process step in the PECVD reactor. Figure 1a–d shows the sample with 1 nm of as-evaporated Sn, after 2 min of H_2_ plasma treatment at 200 °C and 2 min of annealing at 400 °C, after 10 s growth, and after 40 s growth, respectively. It can be seen that as-evaporated Sn forms droplets instead of a continuous layer. The droplets have a density around 9000 droplets/μm^2^, and their diameters range between 1 and 13 nm. The average diameter is around 5.5 nm, and only around 20 % of the droplets have a diameter larger than 8 nm. After annealing at 400 °C, two droplets can merge if they are close enough to each other, as the small droplets inside the white ellipse in Fig. 1a which become one single drop in Fig. 1b. After annealing, the density of droplets decreases to 6600 droplets/μm^2^ and the average diameter increases to 6.3 nm. The changes are mainly due to the merging of droplets.

For Si NW growth catalyzed by Au or Cu, there is an incubation time which can be several tens of seconds [31]. In contrast, when using Sn as a catalyst, NW growth can start within 10 s, presumably due to the low solubility of Si in Sn [32]. Indeed, within 10 s of triggering the SiH_4_ and H_2_ plasma, NWs start to grow, as shown in Fig. 1c. The growth of NWs brings dramatic changes to the droplets. Since the initial distance between droplets is only a few nanometers and the NW length at this growth duration can be 10 to 20 nm, a significant amount of droplets merge, as in the case of the NW indicated by the three dashed white lines in Fig. 1c. The positions of the three roots of this NW correspond to the positions of three droplets indicated by white dots in Fig. 1a, b. It is obvious that these three droplets grow separately and then merge together. In order to illustrate the coalescence due to the NWs growth within the first 10 s, this process has been illustrated with 3D software blender, as shown in Fig. 1e. In this figure, we illustrate how droplets are pushed together and merge during the initial stage of NW growth.

The droplets in Fig. 1a can also catalyze a NW without merging with other droplets, as the droplet and NW indicated by the arrow in Fig. 1a–c. In Fig. 1c, it is difficult to find a NW or droplet with a diameter smaller than 5 nm. This can be due to the low solubility of Si in Sn. When a droplet’s diameter is 5 nm, the number of atoms inside is less than 2500. The solubility of Si in Sn at 400 °C is much less than 0.25 % [32]; thus, the number of Si atoms that can be dissolved inside such a Sn droplet is fewer than 6. In other words, most of the small drops seen in Fig. 1b do not catalyze a NW because they do not have a single Si atom dissolved in them. Therefore, the options for small droplets are to merge with other droplets or to get buried. After the coalescence, the NW can continue its growth; this can be seen in Fig. 1d showing the NWs growing upwards after the coalescence (indicated by the white dashed lines). Droplets can continue coalescing between 10 and 40 s of growth duration, as in the case of the two NWs indicated by the two dashed lines at the bottom part in Fig. 1c, d, which get merged due to NW growth.

The above results are summarized in Fig. 1f where the black squares show the evolution of the droplet density and the red circles show the evolution of the droplet mean diameter. The process time from 0 to 240 s corresponds to the 2 min of H_2_ plasma treatment at 200 °C and 2 min of annealing at 400 °C and from 240 to 250 s corresponds to the first 10 s of the NW growth. With the increase of time, the droplet density keeps on decreasing, while the droplet mean diameter keeps on increasing. Since the droplets’ diameter and density display a much greater change during the 10 s of NW growth than during the 4 min of H_2_ plasma treatment and annealing, it can be concluded that coalescence plays a much more important role than Ostwald ripening.

#### Formation of Straight NWs

**Fig. 2:**
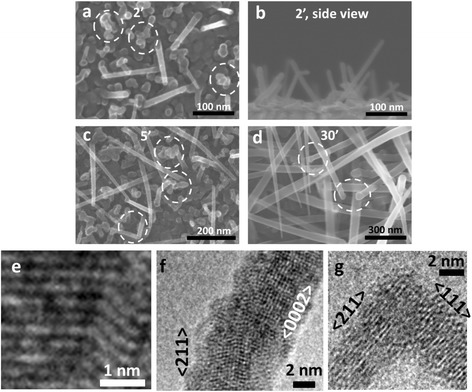
**a**–**d** SEM images of NWs at different growth durations. **a** 2 min; **b** 2 min, side view; **c** 5 min; **d** 30 min. **e**–**f** High-resolution TEM images which show **e** twinning, **f** change of crystalline phase from cubic Si to hexagonal Si, and **g** change of growth direction from <111> to <211>

Let us now look at the NW behavior for longer growth times. At 2 min growth duration, long and straight NWs are clearly observed, as shown in Fig. 2a, b. At this stage, the NW density is around 170 NWs/μm^2^, which is far less than the catalyst droplet density previously mentioned. This is mainly because a large proportion of droplets produce irregularly shaped nanostructures instead of a NW, as indicated by the circle in Fig. 2a. The formation of these irregularly shaped objects might be due to the anisotropic growth breakdown by continuous formation of crystalline defects. The side view SEM image for 2′ growth duration is shown in Fig. 2b. It can be seen that the NWs growth directions are quite random. The NW tip diameter at this growth duration is usually around 10 nm; it is not easy to find a NW with a tip diameter larger than 20 nm, even amongst thousands of NWs. The catalyst droplet size during this initial growth stage is essential for understanding the behavior of the catalyst during the whole growth process. Wetting of the NW sidewalls by catalyst during NW growth has been suggested in the literature [33]. If this phenomenon happens in the present case, the maximum wetting layer length can be estimated by the following equation:

$$ L=2{r}^2/3cn $$

where *r* is the radius of the NW at the base, *c* is the thickness of a monolayer catalyst (*c* ^3^ is the atomic volume of catalyst), and *n* is the number of layers of catalyst on the sidewall. In our case, *c* ≈ 0.30 nm, *r* ≈ 8 nm, *n* = 1 (which means 1 monolayer of Sn coverage), then L is around 140 nm. However, in the present case, the NWs can grow till several micrometers long. The existence of such a wetting layer at a growth condition similar to the present condition is a matter of debate in the literature [24, 29]. Our results suggest that the wetting layer should follow the tips of NW if it exists during the NW growth.

#### NW Growth

As we increase the growth duration, not all the NWs continue their growth along the axial direction. A significant amount of kinking is observed at 5 min growth duration, as indicated by the circles in Fig. 2c. This is mainly caused by crystalline defects such as twinning and change of crystalline phase, as shown in Fig. 2e, f. Indeed, in Fig. 2f, the crystalline phase has changed from diamond cubic Si to hexagonal Si [34, 35]. This is an exciting topic for further investigation that is out of the scope of this paper. NWs can also change growth direction without introducing crystalline defects; as shown in Fig. 2g, the growth direction has changed from <111> to <211>. The new directions may orient the NWs growth toward the substrate or other directions for which it is difficult for NWs to continue growth. NWs usually form irregular shapes after kinking; thus, they lose their wire-like geometry and cannot be considered as NWs. Thus, kinking decreases NW density sharply. When the growth duration increases to 30 min, as shown in Fig. 2d, the NW “forest” becomes crowded because the NWs become long and their diameters increased. As a result, more and more NWs at the bottom of the NW forest get shaded by the NWs on top of them. The shadowing brings two effects. Firstly, it is difficult for the SiH_x_ radicals to reach the hidden NWs. Secondly, these NWs are no longer observable. At this growth duration, as indicated by the circles in Fig. 2d, parts of the NWs start to have a large tip diameter of several tens of nanometers. This is contrast to the situation at 2 min growth time, when it was almost impossible to find a single NW with a tip diameter bigger than 20 nm. The increase in the tip diameter can be explained by the deposition of a-Si:H on the NW tip when there is no Sn catalyst on it (or no enough amount of Sn to continuously catalyze the growth). Therefore, the increase of NW tip diameter can be considered as a sign of the end of the plasma-assisted VLS growth.

#### End of NW Axial Growth Followed by a-Si:H Coating

**Fig. 3:**
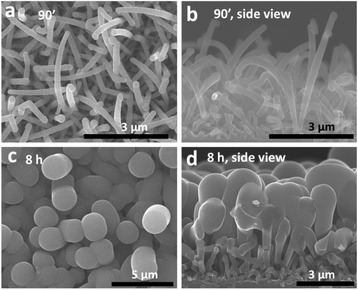
SEM images of NWs at long growth durations: **a** 90 min; **b** 90 min, side view; **c** 8 h; **d** 8 h, side view

When the NW growth duration reaches 90 min, almost all the NWs have a large tip diameter, as shown in Fig. 3a, b. This indicates that all the NWs have eventually run out of catalyst between 30 and 90 min growth duration. As a consequence, there is only additional a-Si:H coating if the growth duration is further increased. This is clearly illustrated in Fig. 3c for 8 h growth duration, where the diameters of the NW tips exceed 1 μm. The NW density as determined from a top view SEM image is less than 1 NW/μm^2^. From the side view SEM image, it can be seen more clearly that NWs touch each other, and most of them merge. This corresponds to standard a-Si:H deposition on a rough surface and the end of NW growth.

### Evolution of Areal Density of Si NWs

**Fig. 4:**
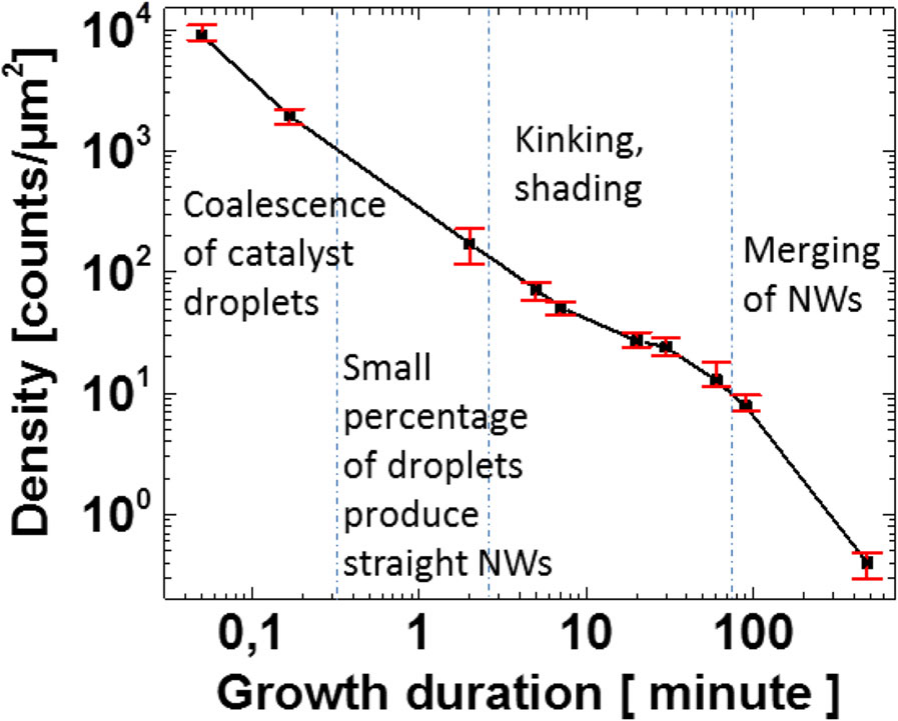
Evolution of the density of catalyst droplets and Si NWs during the 8-h growth process

The evolution of catalyst droplet and NW density is plotted on a log-log plot in Fig. 4. In this figure, the statistics correspond to observable droplets and NWs. The first data point corresponds to the density of catalyst droplets after evaporation. The second data point corresponds to the droplet density at 10 s growth duration. And the rest correspond to the NW density at different growth times. Overall, there is a decrease of density by four orders of magnitude. The decrease can be divided into four stages. The first stage corresponds to the first few tens of seconds of growth, when coalescence of droplets decreases the catalyst density. The second stage corresponds to the first 2 min of growth; at this stage, we have seen that only a small percentage of initial catalyst droplets produce straight NWs. The third stage corresponds to the NW axial growth process, which roughly takes place between 2 and 90 min under our growth conditions. During this stage, the decrease is mainly caused by the kinking and shading of NWs. The last stage (between 90 min and 8 h of growth) corresponds to a phase of standard a-Si:H deposition on the top of NWs; the plasma-assisted VLS process is no longer effective due to the exhaustion of Sn. The increase of diameter makes NWs touch and merge with each other.

### Evolution of Crystallinity

**Fig. 5:**
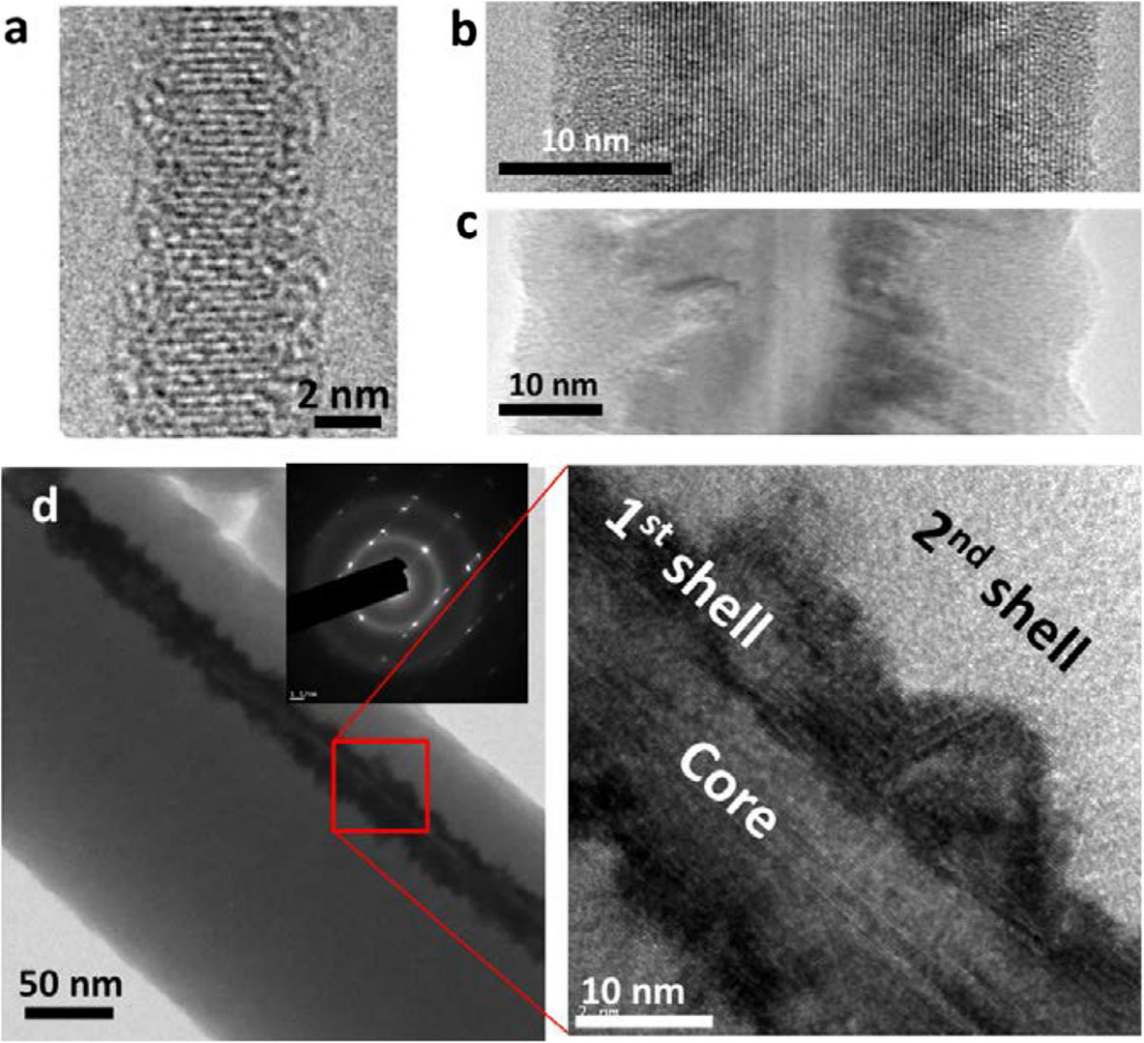
**a** TEM image of a NW with 2 min growth duration. **b** TEM image of the top part of a NW with 30 min growth duration. **c** TEM image of the bottom part of a NW with 30 min growth duration. **d** TEM image of a NW with 90 min growth duration with corresponding diffraction pattern in the *inset*. On the *right* of **d**, we show a zoom of the core double shell structure

We can obtain a clear view on the evolution of the NW crystallinity as a function of growth time through TEM observations. Samples with 2, 30, and 90 min growth duration have been studied by TEM. NWs with short growth duration (~2 min) are usually monocrystalline with an ultrathin (less than 1 nm) amorphous shell on their surface (Fig. 5a). The latter has been characterized as native oxide by electron energy loss spectroscopy (EELS). This oxide is due to the fact that the NWs have been exposed to air before TEM characterization. When the growth duration increases to 30 min, the top part of the NW can still be monocrystalline, as shown in Fig. 5b. The crystalline part almost extends to the NW surface in this TEM image. The middle part of another 30 min-grown NW is shown in Fig. 5c. It can be seen that there is a defective crystalline shell around the core. When the growth duration increases to 90 min, as shown in Fig. 5d, the NW has a monocrystalline core, defective crystalline shell, and a thick a-Si:H shell. Since the core and the two shells are grown in the same experiment without changing conditions, it is hard to imagine the growth changes from crystalline to amorphous with an atomic layer abruptness. In the literature, epitaxial Si growth on crystalline NW core has been achieved at a temperature which is close to ours [36, 37]. Here we propose that the thin defective shell between crystalline core and amorphous layer corresponds to a defective epitaxial growth on the crystalline core [38]. The diffraction pattern in Fig. 5d demonstrates that the defects are twins and the mirror plane of which is essentially the <111>plane that lies parallel to the growth axis. In the literature, crystalline core with nanocrystalline shell structure [24, 29, 39] and crystalline core with amorphous shell structure [25] have been reported. Here, we suggest that with the increase of growth duration, the crystalline structure of NWS evolves from (i) monocrystalline to (ii) monocrystalline core/defective crystalline shell and then to (iii) monocrystalline core/defective crystalline shell/amorphous shell. The interface between crystalline part and amorphous part is not atomically sharp.

**Fig. 6:**
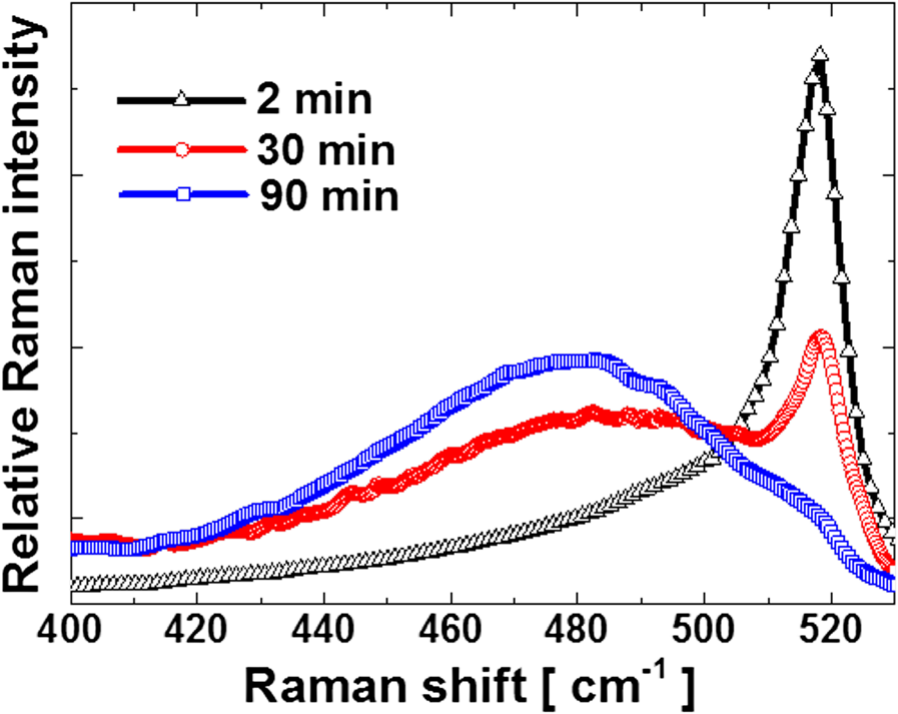
Raman spectra measured on SiNW samples with durations of 2, 30, and 90 min

The evolution of crystallinity has also been studied systematically by Raman spectroscopy for samples with 2, 30, and 90 min growth duration. The diameter of the laser spot is around 1 μm. Thus, the Raman response is that of several tens or hundreds of NWs, depending on the growth duration. The results are shown in Fig. 6. For the sample with 2 min of growth duration, a sharp peak can be observed at around 518 cm^−1^. It indicates that the NWs are almost fully crystalline. There is a 3 cm^−1^ red shift compared with the bulk c-Si Raman peak, which is around 521 cm^−1^. This is mainly because the crystalline NWs have a diameter around 10 nm which produces a red shift of Raman peak [40, 41]. For the sample with 30 min growth duration, the intensity of the crystalline peak at 518 cm^−1^ decreases and a broad a-Si:H peak centered at ~480 cm^−1^ appears. This indicates an increasing fraction of amorphous material, which is mostly due to the a-Si:H deposition on the sidewall of NWs and also on the substrate between the NWs. At 90 min of growth duration, the broad peak at 480 cm^−1^ indicates that the material is mostly amorphous. The very weak shoulder around 520 cm^−1^ shows that the fraction of crystalline material is very small.

### Evolution of NW Morphology

**Fig. 7:**
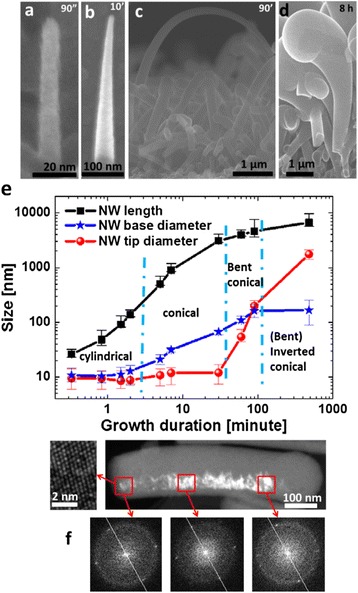
**a**–**d** NW morphology at different growth durations: **a** 90 s; **b** 10 min; **c** 90 min; **d** 8 h. **e** Statistics of NW length, base diameter, and tip diameter at different growth durations. **f** TEM dark-field image of a segment of a bent NW. The image on the *left* is the high-resolution TEM image of the area indicated by the *square*. The three images at *bottom* are FFT of HRTEM images. The *white lines* are reference lines which have been set at the same position. Note that the diffraction spots move from left to right of the line as the FFT rotates clockwise

During the growth process, NWs undergo a strong evolution of morphology from cylindrical to conical, then to bent conical, and lastly to bent inverted conical, as shown in Fig. 7a–d. In Fig. 7e, the black squares, blue stars, and red circles show the evolution of NW length, base diameter, and tip diameter as functions of the growth duration. During the first 30 min of growth, the NW length increases at a rate around 1.5 nm/s, the base diameter increase rate is around 0.03–0.05 nm/s, and the tip diameter remains almost constant. The invariance of the tip diameter can be explained by two reasons. Firstly, the decrease of tip diameter is very small and therefore within the observational error bar. As described before, Sn droplets might not be able to catalyze the growth when their diameter is smaller than 5 nm. So the decrease of the droplets diameter under our growth conditions can only be of a few nanometers since the starting value is around 10 nm. Secondly, the decrease of tip diameter is hidden by another phenomenon: once the Sn is exhausted on the tip, the tip diameter will start to increase due to the coating by a-Si:H. When the coated tip is less than 20 nm in diameter, it is not possible to tell whether the NW axial growth has stopped. This phenomenon increases the average tip diameter and hides the decrease of the tip diameter during NW axial growth.

The evolution from cylindrical to conical shapes can be explained by a-Si:H side wall deposition. Since the radial growth rate is 30–40 times smaller than the axial growth rate, it is not easy to recognize differences in diameter when the NWs are short. At 90 s growth duration, the length of the NW is ~100 nm, and the diameter difference between tip and base is only ~3 nm. Thus, the NWs appear quite cylindrical, as shown in Fig 7a. As the growth duration is increased to 10 min, the NW length ranges from several hundred nanometers to 1 μm, and the base diameters are usually around 40 nm. The base diameter is around four times larger than the tip diameter, and the NWs therefore display a clear conical morphology (Fig 7b).

The NWs axial growth roughly stops between 30 and 90 min due to the exhaustion of Sn. In Fig. 7e, we can see that above 30 min, the NW length does not increase as fast as in the first 30 min. At the same time, the tip diameter increases at a rate higher than the base diameter. As a consequence, the tip diameter can exceed the base diameter at 90 min growth duration. NWs usually bend during this period, as shown in Figs. 3b and 7c. These phenomena can be explained by comparing the mean free path of SiHx radicals with NW size and inter-distance of NWs. The mean free path of the SiHx radicals at the pressures in these experiments can be expressed as [42]:

$$ \lambda =\frac{kT}{\sqrt{2}\pi {d}^2P} $$

where *k*, Boltzman’s constant, *P*, the pressure, *T*, the temperature, and *d*, the molecular diameter. In the present case *P* = 143 Pa, *T* = 673 K, *d* = 0.296 nm. Thus, *λ* has a value around 200 μm. This value is several orders of magnitude larger than the NW length, diameter, and the distance between NWs. When the SiHx radicals arrive at NWs from the top, they can be considered to have a very long and straight trajectory and arrive with a broad angular distribution. Therefore, due to shadowing effects, the tip of the NW receives many more SiHx radicals than the base due to the crowding of NWs, leading to a faster tip diameter growth rate. As the NW diameter increases, the inter-NW distance decreases. It gets more and more difficult for the SiHx radicals to arrive the base of the NWs, and the base diameter stays almost unchanged during 90 min to 8 h growth.

The long SiHx radical mean free path also leads to an asymmetric a-Si:H deposition around the NWs. When the NW is tilted, its upper, plasma-facing side receives more radicals than its lower, substrate-facing side. The dark-field TEM image of Fig. 7f a shows that the a-Si:H shell deposition is not symmetric; the side which has a smaller curvature has thicker a-Si:H deposition. Numerous SEM observations show that the upper side of the bent NWs has a smaller curvature than the lower side, as shown in Fig. 7c, indicating that their upper side has a thicker a-Si:H layer. In Fig. 7f, the high-resolution TEM image shows that the core of the NW is crystalline. The pattern of the fast Fourier transforms (FFTs) of the HRTEM image rotates clockwise as the zone of the FFT is moved from left to right. This indicates that the crystalline core is bent with the same curvature as this segment of the NW. The curvature of the core is around 2*10^5^ m^−1^, and this roughly corresponds to a strain value of 0.5 %. Since a-Si:H can induce stress, the most probable explanation is that the a-Si:H is compressively strained and that this compressively stressed a-Si:H induces a tensile stress onto the crystalline core. Since the upper side of the a-Si:H shell is thicker than the lower side, the tensile stress exerted on the upper side of the core is greater than that on the lower side, and the bending direction is toward the lower side.

### Growth Scenario

**Fig. 8:**
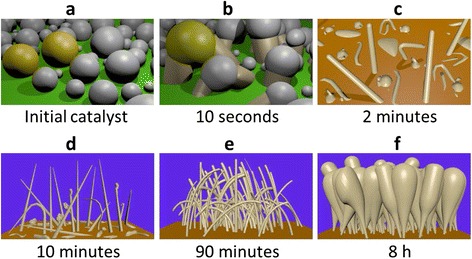
3D illustration of samples at different growth times. **a** as-deposited Sn catalyst; **b** 10 s growth; **c** 2 min growth; **d** 10 min growth; **e** 90 min growth; **f** 8 h growth

Based on the experimental observations presented above, we propose a detailed scenario for the NW growth process. At the very beginning, a discontinuous Sn layer with nominal thickness of 1 nm is evaporated on ZnO:Al substrate. However, instead of forming a continuous layer, the Sn forms droplets which have a mean diameter around 6 nm (Fig 8a). The Sn droplets get oxidized due to their exposure to air between the evaporation and loading into the PECVD reactor. A H_2_ plasma is applied to reduce the Sn oxide into Sn. After that, the Sn droplets are heated to 400 °C and a H_2_ and SiH_4_ plasma is ignited. SiH_x_ radicals produced by the dissociation of silane in the plasma arrive on the Sn droplets, and Si atoms get dissolved into the Sn droplets. As more and more Si atoms get dissolved into the Sn droplets, Si atoms start to precipitate at the interface between the ZnO:Al substrate, the Sn droplet, and the vapor phase. The precipitated Si atoms form an initial nucleus. At the same time, SiH_x_ radicals continue to arrive at Sn droplet surface, diffuse to the nucleus, and form the NW with random growth direction. Since the Sn droplets are only a few nanometers apart, a large proportion of droplets merge during the growth of NWs, as shown in Fig. 8b. A significant proportion of droplets catalyze Si objects with irregular shapes instead of a straight NW. However, a small percentage of droplets catalyze straight monocrystalline (or sometimes twinned crystalline) Si NWs, as shown in Fig. 8c.

These NWs grow rapidly in the axial direction with the help of the Sn catalyst. In the meantime, SiH_x_ radicals also reach the NW sidewalls. These radicals deliver a few nanometers to a few tens of nanometers of radial epitaxial growth before breakdown and the development of an amorphous Si coating on the sidewalls. The radial sidewall growth rate is around 30–40 times smaller than the axial growth rate. The diameter of the NWs at their tip remains almost constant during the growth. Since the base part of the NWs experiences a longer deposition duration, its diameter is larger. This results in a slightly conical geometry for the NWs. During the growth process, there are always NWs that stop growing due to kinking, as shown in Fig. 8d. After a certain growth duration, all the NWs stop axial growth because of the exhaustion of the Sn catalyst. Then the top diameters of these NWs start to increase due to the deposition of a-Si:H on the top part. Since the mean free path of SiH_x_ radicals in the plasma is much bigger than the NW length and the distance between the NWs, it is difficult for the SiH_x_ radicals to arrive at the bottom part of the crowded NW forest. Therefore, the increase of NW diameter at the top part is bigger than that at the bottom part.

When the NW axial direction is not perpendicular to the substrate, there is more a-Si:H deposition on the upper plasma-facing part of the NW sidewall than the lower part. Therefore, the asymmetric compressive stress in the a-Si:H bends the NWs toward the substrate (Fig. 8e). The space between NWs decreases as the diameter of their tops increase, and eventually, the NWs merge. In the end, the structure formed includes a dense bottom part with relatively small diameters, a merged middle part, and a low-density top with relatively big diameters, as shown in Fig. 8f.

## Conclusions

We have carried out a detailed study of the plasma-assisted VLS silicon nanowire growth process by stopping the same growth experiment at different growth times. For the initial growth stage, the evolution of the same droplet of a same sample has been traced from catalyst droplet formation to 40 s of growth. We have studied the evolution of NW density, morphology, and crystallinity during the growth process. Our results show that there is a decrease of density by four orders of magnitude. This can be attributed to (i) the coalescence of the catalyst droplets, (ii) the small percentage of catalysts producing straight NWs, (iii) kinking and shading, and (iv) merging of NWs. We show that NWs undergo a strong evolution in morphology from cylindrical to conical, then to bent-conical, and finally, to bent inverted conical. This is because the radial growth rate is around 30–40 times smaller than axial growth rate. The NW tip diameter and base diameter are too small to be distinguished when the growth duration is short. After the Sn catalyst gets exhausted, sidewall growth rate at the tip is higher than at the base, and the tip diameter can catch up to and eventually exceed the base diameter. The asymmetric stress induced by asymmetric a-Si:H deposition on the upper and lower sidewalls of the NW bends the NWs. We show that at the beginning of the growth, the NW is single crystalline. The sidewall growth firstly results in a few naometers to a few tens of nanometers of defective epitaxial growth before the development of an amorphous Si coating. These observations allow us to propose a full picture of the growth process, as has never been done to our knowledge. The better understanding of the growth process achieved in this research may enable better controlled growth for device applications, in particular solar cells.

## Abbreviations

a-Si:H: Hydrogenated amorphous Si
c-Si: Crystalline Si
EELS: Electron energy loss spectroscopy
HRTEM: High-resolution TEM
FFT: Fast Fourier transform
NW: Nanowire
NWs: Nanowires
PECVD: Plasma-enhanced chemical vapor deposition
sccm: Standard cubic centimeters per minute
SEM: Scanning electron microscopy
TEM: Transmission electron microscope
VLS: Vapor-liquid-solid
ZnO:Al: Aluminum-doped zinc oxide
